# Parallel ensemble of a randomization-based online sequential neural network for classification problems using a frequency criterion

**DOI:** 10.1038/s41598-024-66676-9

**Published:** 2024-07-12

**Authors:** Elkin Gelvez-Almeida, Ricardo J. Barrientos, Karina Vilches-Ponce, Marco Mora

**Affiliations:** 1 Doctorado en Modelamiento Matemático Aplicado, Universidad Católica del Maule, 3480112 Talca, Chile; 2 Laboratory of Technological Research in Pattern Recognition (LITRP), Facultad de Ciencias de la Ingeniería, Universidad Católica del Maule, 3480112 Talca, Chile; 3 Facultad de Ciencias Básicas y Biomédicas, Universidad Simón Bolívar, San José de Cúcuta, 540006 Colombia; 4 Departamento de Computación e Industrias, Facultad de Ciencias de la Ingeniería, Universidad Católica del Maule, 3480112 Talca, Chile

**Keywords:** Computer science, Computational science, Applied mathematics

## Abstract

Randomization-based neural networks have gained wide acceptance in the scientific community owing to the simplicity of their algorithm and generalization capabilities. Random vector functional link (RVFL) networks and their variants are a class of randomization-based neural networks. RVFL networks have shown promising results in classification, regression, and clustering problems. For real-world applications, learning algorithms that can train with new samples over previous results are necessary because of to the constant generation of problems related to large-scale datasets. Various online sequential algorithms, commonly involving an initial learning phase followed by a sequential learning phase, have been proposed to address this issue. This paper presents a training algorithm based on multiple online sequential random vector functional link (OS-RVFL) networks for large-scale databases using a shared memory architecture. The training dataset is distributed among *p* OS-RVFL networks, which are trained in parallel using *p* threads. Subsequently, the test dataset samples are classified using each trained OS-RVFL network. Finally, a frequency criterion is applied to the results obtained from each OS-RVFL network to determine the final classification. Additionally, an equation was derived to reasonably predict the total training time of the proposed algorithm based on the learning time in the initial phase and the time scaling factor compared to the sequential learning phase. The results demonstrate a drastic reduction in training time because of data distribution and an improvement in accuracy because of the adoption of the frequency criterion.

## Introduction

The random vector functional link (RVFL) network is an artificial neural network (ANN) that belongs to the family of randomization-based feed-forward neural networks^1,2^. This algorithm was proposed by Pao et al.^3,4^ and is characterized by direct links from the input layer to the output layer, with randomly assigned weights and biases in the hidden layer. Because of these features, particularly the direct links from the input layer to the output layer, the overall performance of the RVFL network is better than that of other such networks without direct links^5^. The popularity and acceptance of the RVFL network has increased among the scientific community because of the simplicity of the model and its capacity for generalization to classification, regression, and clustering problems. However, the large database of RFVL networks poses a challenge as the training times are considerably longer and high-cost computational architectures are required^6^.

Various architectures and variants of the RVFL network, including RVFL for imbalance learning, kernelized RVFL, RVFL for semisupervised learning, online RVFL, ensemble learning, robust RVFL, ensemble deep RVFL, hybrid RVFL, and deep RVFL, are reported in literature^7^. The focus of the present study was on sequential online algorithms and ensemble models. Liang et al.^8^ proposed a sequential online algorithm for RVFL without direct links. This algorithm was proposed for applications where training data are entered one-by-one or chunk-by-chunk. The algorithm updates the training using new training data and the previous results without utilizing all the accumulated training data. The results showed that these algorithms are faster than other sequential algorithms. Other sequential online algorithms without direct connections in RVFL have been reported in literature. Matias et al.^9^ proposed a sequential online algorithm based on recursive least squares, while Mirza et al.^10^ introduced a voting-based weighted online sequential algorithm for imbalanced multiclass classification. Recently, Gelvez-Almeida et al.^11,12^ proposed a parallel training approach for a set of online sequential algorithms tailored for large-scale datasets using a fingerprint dataset. Additionally, Wibawa et al.^13^ used a model predictive control approach to modify the standard online sequential model. Other recent contributions to online sequential RVFL (OS-RVFL) include the works by Chen and Li^14^, Zhang et al.^15^, Zha et al.^16^, Kale et al.^17^, and Polat et al.^18^.

Several ensemble models have been developed^19^. Lan et al.^20^ introduced an ensemble of OS-RVFL without direct links; this ensemble model was more stable and accurate than the standard online sequential model proposed by Liang et al.^8^ In this ensemble model, multiple online sequential networks are trained, and the average of their outputs is used as the performance of the network. Liu and Wang^21,22^, and Wei et al.^23^ proposed an ensemble-based RVFL network that incorporates cross validation and a criterion based on the norm of network output weights. Zhai et al.^24^ developed an algorithm for integrating the standard OS-RVFL into classification problems with large datasets. Alhamdoosh and Wang^25^ employed RVFLs as the base components and combined them with the negative correlation learning strategy to construct neural network ensembles. Their technique was more effective and efficient than other ensemble techniques. Subsequently, Mirza et al.^26^ presented an ensemble of a subset of OS-RVFL for addressing class imbalance and concept drift. This proposal processes the minority classes with multiple classifiers, while the majority classes are processed in a round-robin fashion. Latter, Ling et al.^27^ proposed an improved ensemble of RVFL networks based on particle swarm optimization with a double optimization strategy, and Huang et al.^28^ proposed a parallel ensemble method based on MapReduce for large-scale learning.

Next, Rakesh and Suganthan^29^ proposed an ensemble of kernel ridge regression using the RVFL network to generate training samples for multiclass classification. Zhang and Suganthan^30^ introduced an efficient co-trained kernel ridge regression method and presented an ensemble of RVFL networks. Li et al.^31^ proposed a parallel one-class approach based on the Bayesian approach. Katuwal and Suganthan^32^ proposed an ensemble of RVFL networks by incorporating additional regularization or randomization through Dropout and DropConnect techniques. Then, Li et al.^33^ proposed a novel ensemble that initializes its base learners using different distributions to enhance their diversity. Huet al.^34^ introduced an adaptive ensemble variant of the RVFL network. Malik et al.^35^ combined the rotation forest and RVFL classifiers into an ensemble method for classification problems. Tanveer et al.^36^ proposed ensemble classifiers with RVFL using multiple SVD models. Shi et al.^37^ proposed deep-learning frameworks based on the RVFL network.

Meanwhile, online sequential algorithms and ensemble models based on RVFL have been used several real-world applications, such as online adaptive humidity monitoring^38^, industrial processes^39^, eye-tracking-based situation awareness recognition^40^, diagnosis of Alzheimer’s disease^41^, short-term electric-load forecasting^42^, landslide displacement prediction^43^, drought index forecasting^44^, turbofan engine direct thrust control^45^, low-resolution real-time face recognition^46^, cross-person and cross-position activity recognition^47^, laminar cooling water supply system for hot rolling mills driven by digital twin for energy-saving^48^, lane-changing control of vehicle platoon^49^, sediment transport in sewer pipes^50^, stock index trend prediction^51^, battery state of health estimation and remaining useful life prediction^52^, etc^53–62^.

Based on the previous works, we present an ensemble of OS-RVFL networks for classification problems with large-scale bases on a frequency criterion (EOS-RVFL-FC). The proposed model involves training several OS-RVFL networks in parallel via multithread computing, classifying the samples tested with all trained OS-RVFL networks, and finally, selecting from the individual results the classes with the highest frequency. This model is more accurate than the original online sequential algorithm. Further, the training time decreases when the training data are distributed and remains constant when they are replicated. To evaluate the efficiency of our proposal, we used a balanced fingerprint dataset. In addition, we used five datasets that have been widely used to validate randomized neural networks algorithms: MNIST, image segmentation, Adult, satellite image, and Mushroom. Four of these datasets are balanced. The main contributions of our article are as follows:

- We proposed a model that leverages the advantages of multithreaded programming to train several OS-RVFL networks in parallel, thus reducing the training time when training data are distributed. A frequency criterion is used to improve accuracy in classification problems.
- We experimentally demonstrated that the proposed method can effectively improve the computational time of the standard OS-RVFL network, increasing the accuracy of the testing data for all the databases. Thus, applying this method in other randomization-based neural networks will be a major scientific contribution.
- We derived an equation that can reasonably estimate the behavior of our model based on the threads to be used. The parameters required are the execution time in the initial phase and its relationship with each chunk of the sequential learning phase, the total training samples, and the training samples in each chunk.

The rest of this paper is organized as follows: Section 2 briefly introduces the preliminary concepts, namely the RVFL network and its sequential online proposal. Section 3 describes our proposed model, including its algorithm and a graphical overview. Section 4 presents the experimental aspects, including a description of the databases, hyperparameter estimation, and the results. Finally, the conclusions and future studies are presented in Section 5.

## Preliminaries

In this section, we present relevant previous works. We provide a brief description of the mathematical frameworks of RVFL networks introduced by Pao et al.^3,4^, as well as the sequential online models proposed by Liang et al.^8^

### Random vector functional link network

**Figure 1:**
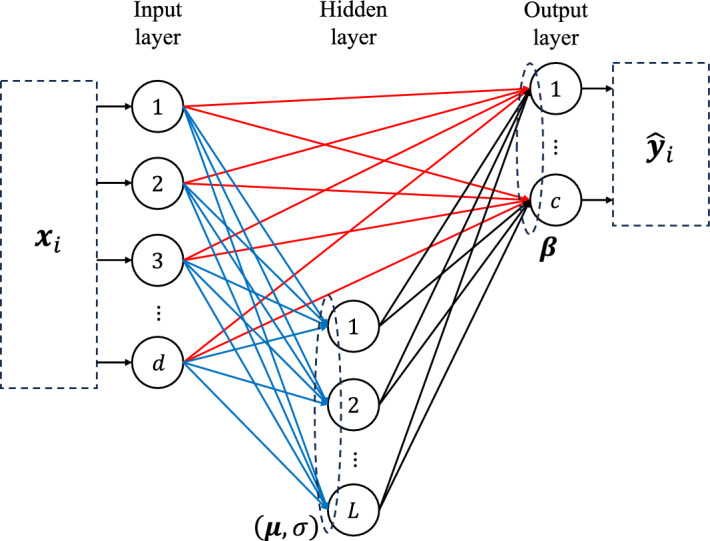
Standard model of the random vector functional link network. The red lines show the connection between the input layer and the output layer; the blue lines represent the connection between the input layer and the hidden layer; the black line represents the connection between the hidden layer and the output layer. The weights $\varvec{\mu }$ and biases $\sigma$ are randomly assigned, while the weights $\varvec{\beta }$ are computed analytically.

RVFL is a single-layer feed-forward neural network that randomly assigns weights and biases to the hidden layer and analytically calculates the weights of the output layer. Let ${\textbf{Z}}$ be an arbitrary training set ${\textbf{Z}} = \{ ({\textbf{x}}_i, {\textbf{y}}_i) | {\textbf{x}}_i \in {\mathbb {R}}^d, {\textbf{y}}_i \in {\mathbb {R}}^c \}$ with $i = 1, \ldots , N$, where ${\textbf{x}}_i$ represents the *i*-th training sample; ${\textbf{y}}_i$, the *i*-th target; *d*, the features of each sample; *c*, the number of classes; and *N*, the total number of samples. In the training process of the standard RVFL network, the three layers are connected as shown in Fig. 1. The input layer and the output layer are connected through randomly assigned weights and biases, while the output layer is connected to the other layers through analytically calculated weights. The training algorithm of the standard RVFL network can be written as follows:

1 $$\begin{aligned} f({\textbf{x}}_i) = \sum _{k=1}^{d} \varvec{\beta }_k {\textbf{x}}_{ik} + \sum _{k=1}^{L} \varvec{\beta }_k \varvec{\theta }(\varvec{\mu }_k \cdot {\textbf{x}}_i + \sigma _k), \end{aligned}$$

where $\varvec{\mu }_k$ and $\sigma _k$ are the *k*-th weights and bias of the hidden layer, respectively; $\varvec{\beta }_k$, is the *k*-th weight of the output layer; $\varvec{\mu }_k \cdot {\textbf{x}}_i$ represents the inner product of $\varvec{\mu }_k$ and ${\textbf{x}}_i$; *L* is the number of neurons in the hidden layer.

The regularized optimization problem for a standard RVFL network with *L* neurons in the hidden layer can be written as follows:

2 $$\begin{aligned} \min _{\varvec{\beta }} || {\textbf{H}} \varvec{\beta } - {\textbf{Y}} ||^2 + \frac{1}{C} || \varvec{\beta } ||^2, \end{aligned}$$

where ${\textbf{H}} = [{\textbf{D}} {\textbf{X}}]$ is the concatenation of hidden features and original features, and *C* is the regularization parameter. Here, ${\textbf{X}} = [{\textbf{x}}_1, {\textbf{x}}_2, \ldots , {\textbf{x}}_N]^T$ is the training dataset, ${\textbf{Y}} = [{\textbf{y}}_1, {\textbf{y}}_2, \ldots , {\textbf{y}}_N]^T$ is the target matrix, and the output matrix of the hidden layer ${\textbf{H}}$ is given as shown below:

3 $$\begin{aligned} {\varvec{D}} = \begin{bmatrix} \varvec{\theta }(\varvec{\mu }_1 {\textbf{x}}_1 + \sigma _1) &{} \cdots &{} \varvec{\theta }(\varvec{\mu }_L {\textbf{x}}_1 + \sigma _L)\\ \vdots &{} \ddots &{} \vdots \\ \varvec{\theta }(\varvec{\mu }_1 {\textbf{x}}_N + \sigma _1) &{} \cdots &{} \varvec{\theta }(\varvec{\mu }_L {\textbf{x}}_N + \sigma _L) \end{bmatrix}_{N \times L} \end{aligned}$$

The output layer weights $\varvec{\beta } = [\varvec{\beta }_1, \varvec{\beta }_2, \ldots , \varvec{\beta }_{(d+L)}]^T$ are calculated analytically from

4 $$\begin{aligned} \varvec{\beta } = {\textbf{H}}^{\dagger } {\textbf{Y}}, \end{aligned}$$

where ${\textbf{H}}^{\dagger }$ is the Moore–Penrose generalized inverse of the ${\textbf{H}}$ matrix. In RVFL networks, the Moore–Penrose generalized inverse matrix of ${\textbf{H}}$ is computed as follows:

5 $$\begin{aligned} {\textbf{H}}^{\dagger } = \left\{ \begin{aligned} \left( {\textbf{H}}^T {\textbf{H}} + \frac{{\textbf{I}}}{C} \right) ^{-1} {\textbf{H}}^T, \quad (d + L) \le N \\ {\textbf{H}}^T \left( {\textbf{H}} {\textbf{H}}^T + \frac{{\textbf{I}}}{C} \right) ^{-1}, \quad (d + L) > N \end{aligned} \right. \end{aligned}$$

where ${\textbf{H}}^T {\textbf{H}}$ and ${\textbf{H}} {\textbf{H}}^T$ are symmetric positive semidefinite matrices and $C > 0$^7^.

### Online sequential random vector functional link

**Figure 2:**
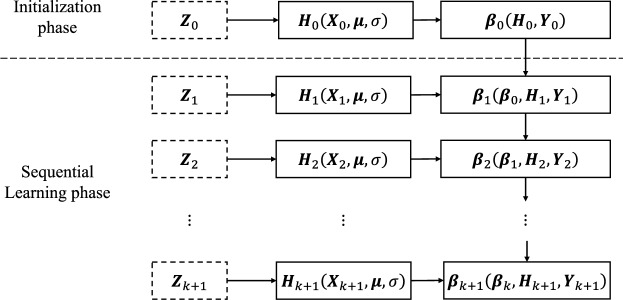
Training algorithm of the standard OS-RVFL network. The weights of the new output layer $\varvec{\beta }_{k+1}$ are updated with the new training data ${\textbf{Z}}_{k+1}$ and the weights $\varvec{\beta }_k$ from the previous output layer.

This algorithm is a variant of the RVFL networks for real-world applications as the data for training are received in a chunk-by-chunk or one-by-one (a special case of chunks) manner^8^. The training algorithm of this model involves an initial phase and a sequential learning phase. Fig. 2 shows a general outline of this algorithm.

#### Initialization phase

Let an initial chunk of training samples ${\textbf{Z}}_0 = \{ ({\textbf{x}}_i, {\textbf{y}}_i) | {\textbf{x}}_i \in {\mathbb {R}}^n, {\textbf{y}}_i \in {\mathbb {R}}^c \}$ with $i = 1, \ldots , N_0$, and *L* neurons in the hidden layer with $L \le N_0$, where $N_0$ is the number of examples in the initial training chunk ${\textbf{Z}}_0$. Thus, random weight $\varvec{\mu }_i$ and bias $\sigma _i$ are assigned. Then, the initial output matrix ${\textbf{H}}_0$ of the hidden layer is calculated. Finally, the initial weights $\varvec{\beta }_0$ of the output layer are computed as follows:

6 $$\begin{aligned} \varvec{\beta }_0 = {\textbf{K}}_0^{-1} {\textbf{H}}_0^T {\textbf{Y}}_0, \end{aligned}$$

where ${\textbf{K}}_0 = {\textbf{H}}_0^T {\textbf{H}}_0 + {\textbf{I}}/{C}$, and ${\textbf{Y}}_0$ is the target matrix of the initial training chunk ${\textbf{Z}}_0$.

#### Sequential learning phase

Let us consider the second chunk of training samples ${\textbf{Z}}_1 = \{ ({\textbf{x}}_i, {\textbf{y}}_i) | {\textbf{x}}_i \in {\mathbb {R}}^n, {\textbf{y}}_i \in {\mathbb {R}}^c \}$ with $i = N_0 + 1, \ldots , N_0 + N_1$. Here, $N_1$ is the number of training samples in the second training chunk ${\textbf{Z}}_1$. Here, the weights of the output layer $\varvec{\beta }_1$ are updated as follows:

7 $$\begin{aligned} \varvec{\beta }_1 = \varvec{\beta }_0 + {\textbf{K}}_1^{-1} {\textbf{H}}_1^T \left( {\textbf{Y}}_1 - {\textbf{H}}_1 \varvec{\beta }_0 \right) , \end{aligned}$$

where $${\textbf{K}}_1 = {\textbf{K}}_0 + {\textbf{H}}_1^T {\textbf{H}}_1$$^8^.

In general, the sequential learning phase presents the $(k + 1)$-th training chunk ${\textbf{Z}}_{k+1} = \{ ({\textbf{x}}_i, {\textbf{y}}_i) | {\textbf{x}}_i \in {\mathbb {R}}^n, {\textbf{y}}_i \in {\mathbb {R}}^c \}$ with $i = (\sum _{j=0}^k N_j) + 1, \ldots , \sum _{j=0}^{k+1} N_j$, where $N_{k+1}$ is the number of training samples in the $(k + 1)$-th training chunk. Then, the partial hidden layer output matrix ${\varvec{H}}_{k + 1}$ for the $(k + 1)$-th training chunk is calculated. Finally, the weights $\varvec{\beta }_{k+1}$ of the output layer are computed using the following equation:

8 $$\begin{aligned} \left\{ \begin{aligned}&{\textbf{P}}_{k+1} = {\textbf{P}}_k - {\textbf{P}}_k {\textbf{H}}_{k+1}^T \left( {\textbf{I}} + {\textbf{H}}_{k+1} {\textbf{P}}_k {\textbf{H}}_{k+1}^T \right) ^{-1} {\textbf{H}}_{k+1} {\textbf{P}}_k\\&\varvec{\beta }_{k+1} = \varvec{\beta }^k + {\textbf{P}}_{k+1} {\textbf{H}}_{k+1}^T \left( {\textbf{T}}_{k+1} - {\textbf{H}}_{k+1}\varvec{\beta }_k \right) \end{aligned}, \right. \end{aligned}$$

where ${\textbf{P}}_k = {\textbf{K}}_k^{-1}$^8^. The sequential learning phase ends when $\varvec{\beta }_{k+1}$ is computed with the last chunk.

## Proposed parallel ensemble method

**Figure 3:**
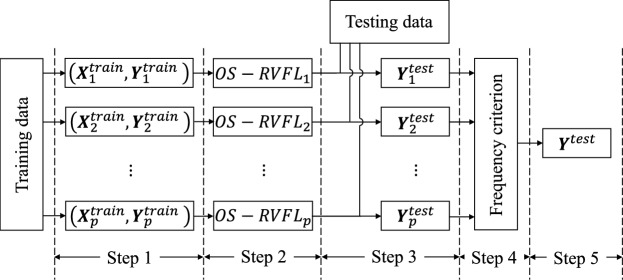
Model of the parallel ensemble of OS-RVFL using a frequency criterion (EOS-RVFL-FC). The criterion involves selecting the label with the highest frequency from among the outputs of each neural network.

Various ensemble-based RVFL models are reported in literature^7^. In this paper, we propose a model that combines the advantages of ensemble models, sequential online algorithms, RVFL networks, and high-performance computing. Our model involves training multiple OS-RVFL networks in parallel by assigning the training phases of each OS-RVFL to a thread through a shared memory architecture. Additionally, we implement data distribution to reduce the training time, considering that we are using a large-scale database. The OS-RVFL version used in this research is the one proposed by Liang et al.^8^, which derives from the RVFL variant without direct link between the input and output layer^63^. Finally, we use a frequency criterion for the final classification of the testing data to consider the results obtained from each neural network. Fig. 3 shows an overall framework of our parallel ensemble proposal. The algorithm for our parallel ensemble proposal is presented as Algorithm 1, which can be summarized in the following steps:

- *Step 1.* The training data are divided into *p* subsets, which are processed in parallel by independent threads. Mathematically, this distribution can be represented as follows:
  9 $$\begin{aligned} \bigcup _{i=1}^{p} {\textbf{Z}}_i = {\textbf{Z}}, \end{aligned}$$
  where ${\textbf{Z}}_i$ denotes each training subset, and ${\textbf{Z}}$ represents the complete training set.
- *Step 2.* Each subset is used to train an individual OS-RVFL network. Each neural network operates independently with its own set of training data and randomly initialized weights and biases.
- *Step 3.* Each OS-RVFL network performs the classification of the same set of test data. As each neural network operates independently, the individual accuracy may vary, leading to potential variation in the results.
- *Step 4.* A frequency criterion is used to analyze the outputs obtained in step 3. The criterion involves selecting the output with the highest occurrence frequency among each neural network result.
- *Step 5.* After applying the frequency criterion in step 4, the final classification is obtained.

This approach is suitable for both distributed and replicated data scenarios. When replicated data are used, each OS-RVFL network is trained using the exactly same training data. Each OS-RVFL network is trained with randomly assigned weights, rendering each network independent and exhibiting varying accuracies, thus making the frequency criterion effective. Replicated data are particularly advantageous when dealing with a limited number of samples. In the realm of computational complexity, OS-RVFL is predominantly shaped by the quantity of examples scrutinized during the training phase^64^. Analogous to standard RVFL and its variants, OS-RVFL equally showcases a low training complexity, notwithstanding the necessity for the algorithm to execute many iterations^65–67^. Our model upholds the computational efficiency of OS-RVFL, as the training of distinct networks is undertaken autonomously and parallel.

**Algorithm 1:**
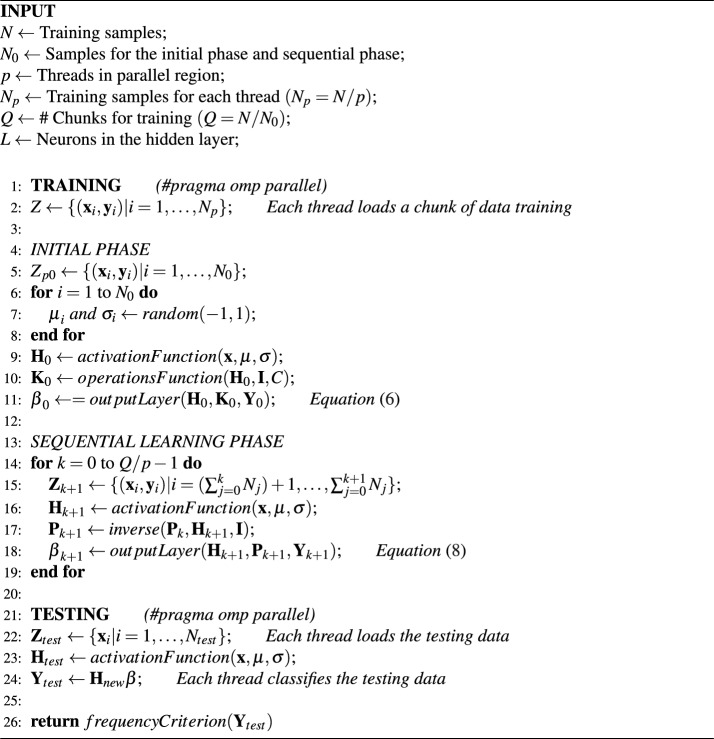
Ensemble of online sequential random vector functional link using a frequency criterion (EOS-RVFL-FC).

## Experiments

In this section, we provide details about the dataset used and outline the hyperparameter estimation procedure. All experiments were conducted on a server equipped with 2$\times$Intel(R) Xeon(R) Gold 6238R CPUs @ 2.20 GHz and with 128 GB RAM. The implementation was written in C++ programming language, using OpenMP to enable parallel processing with shared memory.

### Description of the datasets

**Figure 4:**
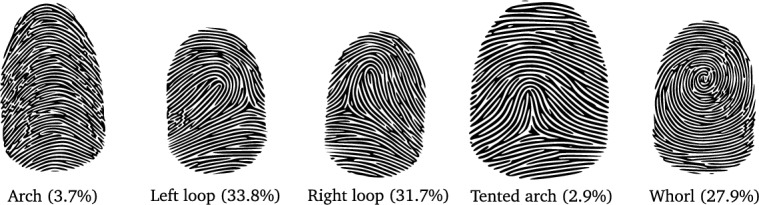
Fingerprint patterns in a population^70^. Fingerprints exhibit distinct patterns that can be classified into five major types: arches, loops, whorls, tented arches, and radial loops. These patterns occur in different proportions within the total population, reflecting the unique distribution and prevalence of each fingerprint type.

In this study, we used a balanced dataset composed of synthetic fingerprint descriptors. The fingerprint descriptors were generated using a feature extractor based on FingerCode, singularities, and pseudo ridges described in a previous work^68^. The dataset has five distinct categories^69^, namely, arch, left loop, right loop, tented arch, and whorl. These categories are shown in Fig. 4. Each category has a different frequency of occurrence within the total population. However, for the purpose of this work, we used a dataset where an equal number of descriptors were available for each class, ensuring a balanced dataset. Each descriptor in the dataset consists of a vector of 202 double-precision type values representing its characteristics, along with the corresponding target. The dataset comprises a total of 210,000 samples, divided into three sets: 200,000 samples for training, 10,000 samples for testing, and an additional 60,000 samples for hyperparameter estimation.

To further evaluate the performance of our model under diverse conditions, we conducted experiments using five publicly available datasets. These datasets were selected to provide a comprehensive evaluation of our approach:

- *MNIST:* This dataset is a widely used benchmark in the field of image classification. It contains a large collection of handwritten digit images, with a training set comprising 60,000 samples and a separate testing set consisting of 10,000 samples. Each image in the dataset is represented as a gray-scale image of dimensions $28 \times 28$ pixels. To facilitate analysis and processing, the images are vectorized, yielding a feature vector with 784 attributes. The MNIST dataset serves as an excellent test bed for evaluating the performance of our proposed model on the task of digit recognition. The distribution of samples varies, with the most represented class having 6265 samples and the least represented class having 5421 samples. Despite this variation, each class is well represented, ensuring a balanced evaluation of the model’s performance across different digits.
- *Image segmentation:* This dataset is a collection of images annotated and labeled for segmentation tasks. Each image in the dataset represents a real-world scene and is accompanied by corresponding segmentation masks, which indicate the pixel-level boundaries of different objects or regions within the image. Similarly to the MNIST dataset, this dataset is well-balanced, containing 300 samples for each class.
- *Adult:* This dataset is a comprehensive collection of demographic and socioeconomic information of individuals. It encompasses a wide range of features, such as age, education level, occupation, marital status, and income. This dataset offers valuable insights into the various factors that influence the socioeconomic dynamics of a population. In contrast to the previously mentioned datasets, this dataset exhibits class imbalance, with one class representing 76.07% of the data and the other class 23.93%. This imbalance poses a significant challenge for classification algorithms, highlighting the need for robust model evaluation and selection strategies.
- *Satellite image:* This image dataset comprises a diverse collection of high-resolution satellite images captured from different regions across the globe. Each image represents a specific area or landscape, showcasing various geographical features such as urban areas, forests, agricultural regions, and more. This dataset offers invaluable insights into land cover analysis, vegetation patterns, and the overall dynamics of the Earth’s surface. The dataset exhibits an unequal distribution of samples among its classes. The three most frequent classes account for 72.8% of the dataset, with each class representing approximately 24.3% of the samples. In contrast, the three least frequent classes comprise 33.1% of the dataset, suggesting a less balanced distribution among these classes.
- *Mushroom:* This dataset is a comprehensive collection of data on diverse species of mushrooms. It encompasses essential features, such as cap shape, cap color, gill size, odor, and habitat. This dataset is popular in the field of classification tasks, specifically in the domain of mushroom identification and toxicity prediction. This dataset serves as a valuable resource for studies on mycology, fungal taxonomy, and the development of intelligent systems for mushroom identification and safety assessment. Importantly, the Mushroom dataset is balanced, ensuring an equitable representation of various mushroom species for classification tasks.

Four of the datasets, including the fingerprint dataset, are perfectly balanced. While our proposal primarily targets balanced databases, we have also included two imbalanced databases to assess the general performance of our approach across varying class distributions. This decision allows for a more comprehensive evaluation of our method’s robustness and effectiveness in handling different dataset characteristics. Table 1 presents an overview of the fundamental characteristics of fingerprints, with detailed information about the additional datasets used in this study.

**Table 1:** Specification of benchmark datasets..

**Dataset**	$\#$Classes	$\#$Attributes	$\#$Training data	$\#$Testing data
Fingerprint^70,71^	5	202	200,000	10,000
Mnist^72^	10	784	60,000	10,000
Image segmentation^73^	7	19	2100	210
Adult^73^	2	14	32,561	16,281
Satellite image^73^	6	36	4430	2000
Mushroom^73^	2	21	7311	813

### Hyperparameter estimation

We conducted an extensive hyperparameter estimation process to optimize the accuracy of OS-RVFL. The hyperparameters under consideration were the number of neurons in the hidden layer and the regularization parameter, denoted as *C*. For this estimation, we used a fingerprint dataset containing 60,000 samples. The dataset was further divided into three subsets: 60% for training (36,000 samples), 20% for validation (12,000 samples), and 20% for testing (12,000 samples).

**Figure 5:**
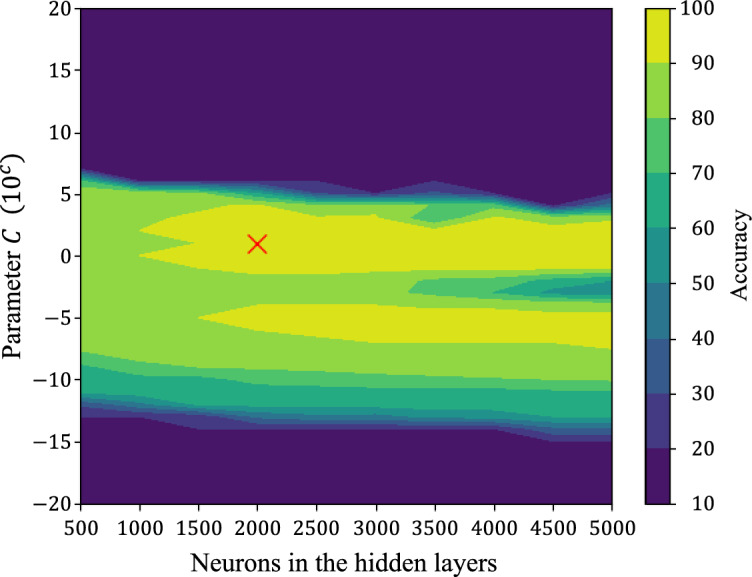
Results of hyperparameter tuning in an OS-RVFL neural network^11^. The accuracy improves when the regularization parameter is between $10^{-10}$ and $10^{10}$, while there is no significant change when the number of neurons in the hidden layer are increased (36,000 samples for training, 12,000 for validation, 12,000 for testing, and sigmoid activation function).

With regard to the hidden-layer neurons, we performed training experiments using 500–5000 neurons, in increments of 500 neurons in each experiment. Further, we explored a wide range of the *C* parameter, from $10^{-20}$ to $10^{20}$, incrementing the exponent by 1. In each phase of OS-RVFL, we used 9,000 samples for both the initial phase and each subsequent chunk in the sequential learning phase. Fig. 5 shows the results obtained for the different combinations of hyperparameters. The accuracy improved remarkably when the regularization parameter *C* was in the range of $10^{-10}$–$10^{10}$. Based on these results, we selected 2000 neurons in the hidden layer and set $C = 10$ as the regularization parameter for the experiments conducted in this work.

For the public datasets, we used the entire dataset to estimate the hyperparameters. We conducted experiments ranging from 100 neurons to 3,000 neurons in the hidden layer, with increments of 100 neurons in each experiment. Notably, each dataset achieved the highest accuracy with a different number of neurons in the hidden layer (see Table 1). By following the approach of previous authors^8,20^, we used $L+100$ samples for the initial phase and each subsequent chunk of the sequential learning phase to ensure effective training.

### Result using fingerprint dataset

We conducted a comparative analysis between the results obtained using OS-RVFL and our distributed model using the fingerprint dataset described in Table 1. For comparison, we evaluated the training time, training accuracy, and testing accuracy as performance metrics (see Table 2). Additionally, we analyzed the speed-up and efficiency of our algorithm using the distributed training data. The speed-up was calculated as follows:

10 $$\begin{aligned} S(p) = \frac{Time(1)}{Time(p)}, \end{aligned}$$

where *S*(*p*) is the speed-up with *p* threads, and *Time*(1) is the training time with 1 thread and *Time*(*p*) with *p* threads. Next, we derived the corresponding efficiency $E(p)=S(p)/p$. In our experiments, we used the sigmoid activation function and set the number of neurons in the hidden layer to 2000, with $C=10$ as the regularization parameter. These configurations were selected based on the results of the previous hyperparameter estimation.

The results in Table 2 demonstrate that the training accuracy of EOS-RVFL-FC-D is comparable to that of the standard OS-RVFL network. However, the testing accuracy improves with an increase in the number of threads. The highest testing accuracy achieved by EOS-RVFL-FC-D was 93.05% when using 10 threads. By applying the frequency criterion, the accuracy was further enhanced, while the training time decreased drastically. Furthermore, the training time of the EOS-RVFL-FC-D decreased as the number of threads increased as the workload was distributed across the available threads.

**Table 2:** Performance of our model with fingerprint dataset using the distributed (EOS-RVFL-FC-D) dataset (200,000 samples training, sigmoid function, 2000 neurons in the hidden layer, and $C = 10$ as the regularization parameter).

#Threads	Training time (seg)	Training accuracy (%)	Testing accuracy (%)	Speed-up	Efficiency
1	74,235	92.07	92.06	–	–
2	37,361	92.28	92.00	1.99	0.99
3	22,675	92.50	92.65	3.27	1.09
4	15,008	92.61	92.72	4.95	1.24
5	12,337	92.71	92.89	6.02	1.20
6	10,947	92.82	92.80	6.78	1.13
7	8259	92.95	92.90	8.99	1.28
8	6382	93.13	92.90	11.63	1.45
9	5129	93.16	92.69	14.47	1.61
10	4685	93.40	93.05	15.85	1.58

**Figure 6:**
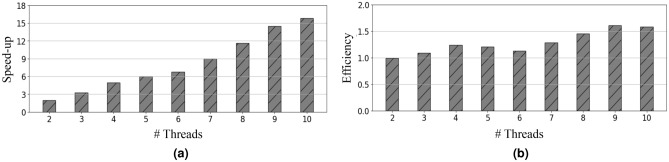
(**a**) Speed-up and (**b**) efficiency of our model with regard to the training time when using a distributed (EOS-RVFL-FC-D) dataset (200,000 samples for training; sigmoid function activation; 2000 neurons in the hidden layer; and $C = 10$ as the regularization parameter). The speed-up was calculated over the standard OS-RVFL network using a single thread.

**Figure 7:**
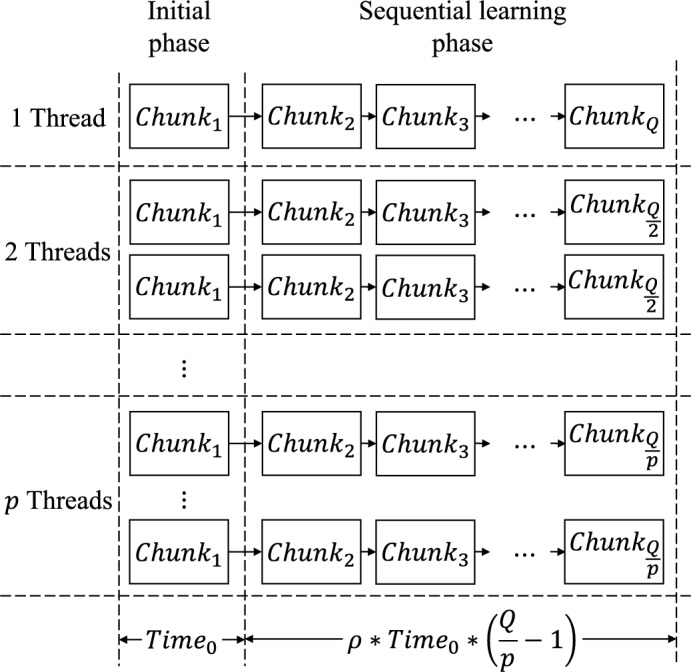
Graphical representation showing the distribution of the dataset and the training time in relation to the number of threads utilized for parallel training. In this graph, *Q* represents the number of chunks used for training; *p* denotes the number of threads; and $\rho$ represents the difference in computation time between $\varvec{\beta }^0$ and $\varvec{\beta }_{k+1}$.

Meanwhile, Fig. 6 shows the speed-up and efficiency of our parallel algorithm in comparison to the sequential version and provides a comprehensive overview of the results. Fig. 6 depicts the noteworthy observations obtained for EOS-RVFL-FC-D. The speed-up exceeds the number of threads (as shown in Fig. 6a), and the efficiency surpasses one (as depicted in Fig. 6b). This phenomenon is attributed to the distribution of samples as the threads increase, allowing each thread to be trained on a smaller subset of samples. Additionally, our experimental results reveal that the computation of $\varvec{\beta }_0$ is approximately 8.8 times faster than in the case of the sequential learning phase $\varvec{\beta }_{k+1}$. A graphical representation illustrating this behavior is shown in Fig. 7. The observed behavior can be effectively modeled using the following equation:

11 $$\begin{aligned} Time(p) = Time_0 + \rho Time_0 \left( \frac{Q}{p} -1 \right) , \end{aligned}$$

where $Time_0$ is the computation time of $\varvec{\beta }_0$ with the $chunk_1$; $\rho$ is the difference in computation time between $\varvec{\beta }^0$ and $\varvec{\beta }^{k+1}$; *Q* represents the chunks for training; and *p* indicates the number of threads used for parallel training with $p \in {\mathbb {N}}$ and $p = 1, \ldots , Q$.

### Results using a large-scale dataset

We conducted experiments with a large-scale fingerprint database. The database consists of 1,000,000 training samples, 10,000 testing samples, and the same characteristics as those listed in Table 1 (classes and attributes). These experiments used 6–46 threads with a power-of-two growth. Table 3 lists the results related to training time, training accuracy, and testing accuracy. Additionally, we compared the speed-up and efficiency of the proposed network to those of the standard OS-RVFL neural network. The results demonstrate that the testing accuracy increases as the number of threads increases, reaching up to 93.19% for 48 threads. Although the improvement in accuracy is not significant, the converse is true for the training time. As shown in Table 3, the training time decreases significantly when 48 threads are used.

**Table 3:** Performance comparison of our proposal with fingerprint dataset using the distributed (EOS-RVFL-FC-D) dataset (1,000,000 samples training, sigmoid function, 2000 neurons in the hidden layer, and $C = 10$ as the regularization parameter).

#threads	Training time (seg)	Training accuracy (%)	Testing accuracy (%)	Speed-up	Efficiency
1	479,981	91.96	92.24	–	–
6	91,054	92.22	92.91	5.27	0.88
12	47,065	91.82	93.09	10.20	0,85
24	23,098	92.71	93.14	20.78	0.87
48	7628	93.38	93.19	62.92	1.31

**Figure 8:**
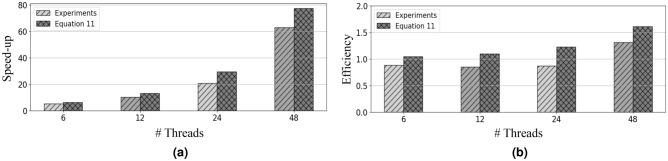
(**a**) Speed-up and (**b**) efficiency of our proposal for training time using distributed (EOS-RVFL-FC-D) dataset (1,000,000 samples training, sigmoid function, 2000 neurons in the hidden layer, and $C = 10$ as the regularization parameter). The speed-up was calculated over the OS-RVFL using a single thread.

Furthermore, we compared the experimental results and those obtained using (11). The parameters employed in (11) were computed based on preceding experiments summarized in Table 2 and Fig. 6. The computation time “$Time_0$”, associated with “$Chunk_1$,” was measured to be 494 seconds, while the time difference $\rho$ between $Time_0$ and the other *Time* values was found to be $8.8\times$. Here, *Q* represents the ratio of *N* to $N_0$, where *N* corresponds to the total samples present in the dataset, and $N_0$ represents the number of samples allocated to each training Chunk. Further, *p* denotes the number of threads employed during the parallel training. Fig. 8 shows a comparison of the speed-up and efficiency between the experimental results and the results using (11).

The speed-up (Fig. 8a) and efficiency (Fig. 8b) achieved by our distributed proposal surpass those of a conventional parallel algorithm, considering the characteristics of sequential training depicted in Fig. 7. These results highlight the effectiveness of our proposal in terms of training time. Furthermore, the outcomes obtained using (11) agree closely with our experimental results. By using the parameters specified in (11), we can estimate the behavior of our distributed proposal when using multiple threads for parallel training. Notably, the accuracy of (11) can be further enhanced by incorporating the behaviors of other factors, such as hardware considerations.

### Results using the replicated dataset

The experimental results we obtained so far can be achieved when working with databases that have sufficient samples to distribute across multiple threads. However, when the database has a limited number of training samples, the frequency criterion proposed herein can be applied by replicating the training samples in each thread (EOS-RVFL-FC-R). To test this approach with replicated data, we adopted widely used public databases to evaluate artificial intelligence algorithms, particularly neural networks for classification problems (see Table 1). It is important to highlight that each OS-RVFL network trains with randomly assigned weights, ensuring that each network is independent. This property guarantees that the frequency criterion remains effective even when training with a replicated database. As a result, the diversity among the independently trained networks contributes to robustness in the final classification decision, as it accounts for different perspectives captured by each network. Table 4 Table 4 lists the results of training time, training accuracy, and testing accuracy, obtained with our proposed method with replicated data. In these experiments, we used 10 threads, while the number of neurons in the hidden layer was obtained from the literature.

**Table 4:** Performance comparison between OS-RVFL against our replicated (EOS-RVFL-FC-R) proposal using different datasets and 10 threads (sigmoid function in the hidden layer).

Dataset	$\#$Neurons	Algorithm	Training	Accuracy	
			Time (seg)	Training	Testing
Mnist	900	OS-RVFL	2,238	93.30	93.09
		EOS-RVFL-FC-R	2,295	93.22	94.68
Image segmentation	600	OS-RVFL	7.03	94.87	94.67
		EOS-RVFL-FC-R	7.04	94.93	96.38
Adult	2500	OS-RVFL	9,105	88.34	88.22
		EOS-RVFL-FC-R	9,082	88.35	88.27
Satellite image	1000	OS-RVFL	73.60	94.34	89.39
		EOS-RVFL-FC-R	70.50	94.45	89.81
Mushroom	300	OS-RVFL	7.73	99.36	99.32
		EOS-RVFL-FC-R	7.73	99.36	99.77

With regard to the training time, we can see from Table 4 that more time is required when the database has a larger number of training samples. However, when the number of samples is very small, the difference in training time is insignificant, as seen in the case of the Mushroom and Image Segmentation datasets. Meanwhile, the accuracy improves in all databases, particularly in the MNIST and Image Segmentation datasets. In the other databases, the improvement in accuracy is minimal. Overall, in applications with small databases, our proposal can enhance accuracy without drastically affecting the training time. However, when the number of samples is larger, the increase in accuracy does not justify the increase in training time. In these cases, it is preferable to evaluate the distribution of samples across multiple threads.

To compare our model with other ensemble OS-RVFL models reported in the literature, we compare the reported results with ours. The results presented by Lan et al.^20^, Liu et al.^22^, and Wei et al.^23^ show that their ensemble models increase accuracy in a similar range to ours. However, the training time significantly increases compared to standard OS-RVFL as the number of networks increases. Results presented by Huang et al.^28^ show a considerable increase in training time, even though their approach is parallel and based on MapReduce. Among the databases used by the authors are Mnist, Image Segmentation, Adult, and Satellite Image, making their results comparable to ours. Therefore, our proposal offers significant advantages over these models, especially concerning training time.

## Conclusions

In this paper, we introduce a frequency criterion in a parallel ensemble algorithm for sequential online RVFL network (OS-RVFL-FC) in large-scale classification problems. We validated our proposed network using a synthetic fingerprint database and five widely used public databases. The parallel ensemble approach involves training multiple OS-RVFL networks by distributing or replicating the database samples and then applying a frequency criterion to the outputs of all the neural networks. The frequency criterion selects the most frequent output among the results obtained from all OS-RVFL networks. We used two methodologies: (1) distributed samples (EOS-RVFL-FC-D) for large-scale databases, and (2) replicated samples (EOS-RVFL-FC-R) for small-scale databases.

The results with EOS-RVFL-FC-D demonstrate that the accuracy increases when trained with multiple threads, while the training time significantly decreases; the achieved speed-up and efficiency exceed those of a conventional parallel program. This improvement originates from the substantial difference between the execution time of the initial phase and the sequential learning with each chunk. Considering this fact, we introduced an equation that can reasonably predict the speed-up and efficiency of our proposal based on the execution time in the initial phase, its relationship with the sequential learning in each chunk, the total number of training samples, and the size of the chunks in both phases.

With regard to EOS-RVFL-FC-R, the results demonstrate that the accuracy increases for all databases, though the improvement is drastically small in some cases. The difference in training time is negligible when the databases have few samples. However, this difference becomes significantly large as the size of the database increases, making the method impractical for large-scale databases. In general, the proposed model with distributed data is suitable for large-scale databases as it significantly reduces the training time as the number of threads increases. On the other hand, for small databases, the proposed model with replicated data can improve the overall accuracy of the neural network. However, when the number of samples is larger, it is more viable to consider the proposed model with distributed data.

In future work, we will continue investigating ensemble methods in randomization-based online sequential neural networks to further improve the accuracy and training time. We plan to include more datasets with imbalanced class distributions to evaluate the effectiveness of future proposals under such conditions. Additionally, we believe it is important to implement this proposed methodology in real-world applications, considering the substantial reduction in training time. Furthermore, we will continue to work on proposals that incorporate statistical improvements in the frequency criterion.

## Data Availability

The public datasets used and/or analyzed during the current study are available in the UCI Machine Learning Repository, [https://archive.ics.uci.edu/datasets]. The fingerprint datasets used and/or analyzed during the current study are available from the corresponding author on reasonable request.
